# Serum Complement C3f and Fibrinopeptide A Are Potential Novel Diagnostic Biomarkers for Non-Alcoholic Fatty Liver Disease: A Study in Qingdao Twins

**DOI:** 10.1371/journal.pone.0108132

**Published:** 2014-09-24

**Authors:** Yong-Ning Xin, Ning Geng, Zhong-Hua Lin, Ya-Zhou Cui, Hai-Ping Duan, Mei Zhang, Shi-Ying Xuan

**Affiliations:** 1 Qingdao Municipal Hospital, Qingdao, Shandong Province, PR China; 2 School of Medicine, Qingdao University, Qingdao, Shandong Province, PR China; 3 Key Laboratory of Ministry of Health for Biotech-Drug, Key Laboratory for Modern Medicine and Technology of Shandong Province, Shandong Medicinal Biotechnology Center, Shandong Academy of Medical Sciences, Jinan, PR China; 4 Qingdao Municipal Centers for Disease Control and Prevention, Qingdao, Shandong Province, PR China; Kaohsiung Medical University Hospital, Kaohsiung Medical University, Taiwan

## Abstract

**Aims:**

To compare the different serum peptidome patterns between twins with and without non-alcoholic fatty liver disease (NAFLD) in order to help understand the pathogenesis of NAFLD and to identify potential diagnostic and therapeutic targets.

**Methods:**

The peptidomics patterns of 63 cases with NAFLD were compared with their twin healthy controls in Qingdao, China. Peptides between 800Da and 3500Da were captured and concentrated using C18 reversed-phase columns, followed by MALDI-TOF mass spectrometry. The sequences of peptides associated with NAFLD were further identified by MALDI-TOF-TOF. Further validation studies were conducted. One hundred additional serum samples were detected by commercially available ELISA kits to calculate the concentrations of complement C3f and fibrinopeptide A, respectively. The differences of these two peptides in the NAFLD and control groups were compared using SPSS 17.0, respectively.

**Results:**

Compared with healthy controls, eleven peaks (861.1, 877.07, 904.5, 1206.57, 1350.64, 1518.7, 1690.9, 1777.94, 2931.29, 3190.4, 3261.4) were up-regulated and 7 peaks (942.44, 1020.47, 1060.06, 1211.7, 1263.63, 1449.76, 2768.3) were down-regulated in the NAFLD group. Two peptides derived from complement C3f and fibrinopeptide A, respectively, had the highest ROC values indistinguishing NAFLD cases from their normal controls. In the validation group, the concentrations of complement C3f and fibrinopeptide A (1466.929±78.306 pg/ml, 4.189±0.326 ng/ml, respevtively) in NAFLD group was higher than in control group (complement C3f 1159.357±99.624 pg/ml, FPA 3.039±0.483 ng/ml; P<0.05).

**Conclusions:**

In this study, we established apeptidomics pattern that could help distinguish NAFLD patients from their twin controls. The differently-regulated peptides identified in our study may be potential diagnostic markers or therapeutic targets for NAFLD.

## Introduction

Nonalcoholic fatty liver disease (NAFLD) is a cause of chronic liver disease that has gained increasing recognition over the past decade. The condition most commonly associated with NAFLD is chronic over nutrition with consequent obesity [1]–[3] and, often, insulin resistance [4], [5]. NAFLD progresses to cirrhosis within 10 years in approximately 5% of patients and has been projected to become the most common indication for liver transplantation in the next 10–20 years [6]–[9]. The biological basis for the wide histological spectrum that occurs in NAFLD remains poorly defined.

New genomic and proteomic technologies have the potential to understand the complex pathogenic mechanisms of NAFLD [10]–[15]. For example, simultaneous assessment of gene expression in a large number of genes and protein peaks can help identify molecular pathways that are active in NAFLD [11]–[16]. Proteomic analyses have been limited by poor sensitivity in detecting low-abundance proteins as well as difficulties with maintaining sample stability and data management. Recent advances in protein separation and improvements in both detection and identification of peptides and proteins have facilitated detailed characterization of complex biological samples, including the liver domain. Proteomic analysis may identify hepatic proteins that contribute to the development of nonalcoholic steatohepatitis (NASH) with progressive fibrosis. As reported by Muir et al. [17], novel candidate targets for hepatocellular carcinoma (HCC) were identified by applying proteomic profiling approaches in PTEN-null NASH liver and tumor samples. Furthermore, differential abundance of hepatic proteins can be confirmed and further studied in a second phase of experiments, including immunohistochemical analysis of samples and a tandem mass spectrometric (MS/MS) approach. Simultaneous analysis of the relative expression of a large number of proteins in a sensitive manner may help to determine the relative expression of hepatic proteins across the histologic spectrum of NAFLD.

In our study, we used the above-mentioned advanced proteomic technologies and sample selection based on disease-discordant monozygotic twins to identify NAFLD diagnostic markers. A matched co-twin analysis is a powerful method to detect biomarker profiles for medical conditions as compared with traditional case–control studies [18]. Considering the subtle distinction in protein levels and the influence of genetic variation among experimental and control subjects [19], a disease-discordant monozygotic twin study is a highly efficient way to mitigate the potentially confounding effects of human genetic polymorphisms [20]. In the present study, we utilized this combined technological and clinical approach to find possible novel diagnostic markers of NAFLD.

## Materials and Methods

### 1 Patients selection criteria

#### 1.1. Ethics Statement

All of the subjects understood the study procedures and signed the informed consents. This study protocol was approved by the Ethics Committee of Qingdao Municipal Hospital and was in compliance with the Helsinki Declaration.

**1.2.** A total of 178 pairs of twins with a mean age of 40 were enrolled in 5 districts in the Qingdao municipality. From this sample, 63 pairs of eligible adult twins were recruited from the Qingdao Twin Registry [21]. Zygosity of all same-sex twin pairs was determined by 16 multiple short tandem repeats [22] at the central laboratory in the Qingdao Blood Bank. Between each pair, one was diagnosed with fatty liver disease (disease group, n =  63) and the other was considered the control (control group, n = 63). Subjects were excluded as follows: history of viral hepatitis, autoimmune hepatitis, and other forms of chronic liver disease including hepatic injury caused by substance abuse; and a current or past history of consumption of more than 20 g of alcohol daily. Moreover a twin-pair was excluded if one of the co-twins declined participation.

In our study, the disease group fulfilled the diagnostic criteria of NAFLD as defined by the Chinese Liver Disease Association [23]. Weight and height were measured with the subject in lightweight clothes with their shoes removed. Weight was measured using a standing beam scale to the nearest 0.1 kg, and height was measured using a vertical scale with a horizontal moving headboard to the nearest 0.1 cm. Body mass index (BMI) was calculated as weight (kg) divided by square of height (m). Waist circumference (cm) was measured on a standing subject using a soft tape midway between the lowest rib and iliac crest to the nearest cm. Hip circumference was measured over the widest part of the gluteal region to the nearest cm, and the waist/hip ratio (WHR) was calculated as well. Blood pressure was measured using standard procedures. Abdominal (hepatic) liver ultrasound was performed in all the subjects. No liver biopsies were obtained in the frame of the present study.

### 2 Blood sampling management

Fasting blood samples were obtained, and supernatants were extracted after 2 rounds of centrifugations at 1,000 g for 5 min and at 12000 g for 5 min, respectively. Then, 500 µlof supernatant of each sample was sub-packaged into collecting pipes and frozen at –80°C until further use. No serum samples were submitted to repeat freeze-thawing more than twice. Liver function tests included serum transaminases (AST: asparate aminotransferase and ALT: alanine aminotransferase), Gamma glutamyl transpeptidase (GGT), and prothrombin index. Serum glucose was also measured using a Semi-automatic Analyzer (Hitachi 7600).

### 3 Affinity Chromatography

To abstract and purify the target peptides from the supernatant, different affinity chromatography methods were conducted, including ZipTip with C18 resin, ZipTip with C4 resin and PepClean C-18 Spin Columns. All processes were according to the manufacturer's protocols. After analyzing the samples via Peptide Mass Fingerprinting (PMF), we found that ZipTip with C18 resin was the most efficient method.

### 4 PMF

An aliquot of extracts (0.3 µl) was mixed with saturated alpha-cyano-4-hydroxy-cinnamic acid (CHCA) matrix, deposited on the MALDI plate, and air-dried at room temperature. To identify the most discriminating serum peptide profiles between subjects with and without NAFLD, we used MALDI-TOF MS (ABI, 4700 TOF/TOF) with the reflective mode. Introducing the batch mode, the mass spectra of peptides was generated at the 800–3500 Da optimization range by using an average of 1000 laser shots at a laser intensity of 5000 arbitrary units. Angiotensin I (Molecular Weight: 1296), Glu1-Fibrinopeptide B (Molecular Weight: 1570), ACTH (clip 1–17) (Molecular Weight: 2093) and ACTH (clip 18–39) (Molecular Weight: 2466) were used as the standard substances to normalize and minimize the error under 10 ppm.

### 5 Bioinformationanalysisof the serum peptide profiles

#### 5.1 Data Mining

All of the preliminary spectrograms were analyzed with Data Explorer software, and the data were output as a peak trace (ASCII format) with monoisotopic peaks and the intensity of the detected peptides. Each crest value represented a free peptide shown with the relative intensity versus the mass-to-charge ratio (m/z) values.

#### 5.2 Screening

The Metalab bioinformatics software package was used to compare the mean intensity of two groups (disease and control). Each isotopic peak and discriminatory peptide peaks were selected. A Mann-Whitney U test was conducted to further validate the significant difference of specific peptide peaks between the two groups. A p<0.01 was considered statistically significant. MSight software (Swiss Institute of Bioinformatics, Switzerland) was employed to construct the figure of discriminatory peaks.

#### 5.3 Systemic Cluster, Principal Component Analysis (PCA) and Receiver Operator Characteristic (ROC) Curve

SPAA 16.5 was used to normalize the discriminatory peak intensity of each peptide and to subsequently analyze the PCA based on the normalized data. To demonstrate the intensity of splitting, thermography and dendrograms were conducted via systemic cluster analysis with Cluster 3.0 software (Version 1.33, University of Tokyo, Human Genome Center). The concrete cluster method was performed using Centroid linkage analysis. The discriminatory peptide diagnostic value for the diagnosis of NAFLD was assessed by determining the area under the curve (AUC) with ROC.

### 6 Identification of the discriminatory peptides

To trace the sources of discriminatory peptides and the specific amino acid sequences, second order mass spectrography was conducted using MALDI- TOF- TOF(ABI 4700) based on the data from PMF analysis. The mass spectrum values were set as follows: reflective mode; batch mode; laser intensity: 6800; and laser shots: 2000 times. The MS/MS fragment data were exported as Sequest files and were used for database searches with the MASCOT search engine using the GSP Explore software (ABI. Inc). The parameters were set according to the following list (Table 1).

**Table 1:** The parameters of database searches with the MASCOT search engine using the GSP Explore software.

Detabase	Swiss-Prot
Type of search	MS/MS Ion Search
Enzyme	No
Variable modifications	Carbamidomethyl (C),Oxidation (M)
Mass values	Monoisotopic
Protein Mass	Unrestricted
Peptide Mass Tolerance	±0.15 Da
Fragment Mass Tolerance	±0.3 Da
Instrument type	Default

## Results

### 1 Patient Demographics and Clinical Data

Table 2 presents the clinical, biochemical and ultrasound data observed in the studied population. It should be emphasized that no environmental factors could be found to explain the differences between the twins with and without NAFLD. The clinical data(BMI, waist circumference and WHR) and the biochemical data(AST, ALT, GGT and total bilirubin) were statistically significantly different between the 2 groups (P<0.01). There was no difference in the prothrombin index (p>0.05).

**Table 2:** Clinical and liver features of the study participants and the validation participants.

Category	Subcategory	study participants		validation participants	
		NAFLD(n = 63)	control(n = 63)	NAFLD(n = 50)	control(n = 50)
Age (years)		40.2±12.2	40.2±12.2	42.4±7.2	43.4±8.1
BMI (kg/m^2^)		33.1±5.9	22.2±3.8	30.1±1.6	24.3±2.0
waist circumference (cm)		92.0±12.8	78±10.5	97.3±7.3	82.0±7.5
WHR		0.93±0.08	0.76±0.05	0.92±0.05	0.86±0.06
Comorbidities	Hypertension (%)	61	10	42	9
	Diabetes mellitus (%)	46	0	28	2
	Dyslipidemia (%)	43	13	35	11
Liver Function Tests	AST (U/L)	64.6±38.7	31.5±9.3	69.4±11.5	28.7±11.4
	ALT (U/L)	73.2±19.1	27.3±8.7	72.0±9.5	24.6±5.5
	GGT (U/L)	79.3±18.9	34.6±7.5	68.6±7.6	27.1±12.4
	Total bilirubin (mg/dL)	0.8±0.2	0.4±0.3	1.3±0.4	0.8±0.2
	Prothrombin index(%)	82.6±13.8	84.2±15.8	83.3±1.5	83.5±2.3
Steatosis (ultrasound)		63	0	50	0

Values are expressed as mean±SD. BMI, body mass index; WHR, waist/hip ratio; AST, aspartate aminotransferase; ALT, alanine aminotransferase; ALP, alkaline phosphatase;GGT, gammaglutamyltranspeptidase; US: ultrasound examination.

### 2 Extraction and purification of serum low molecular weight free polypeptides

**Figure 1:**
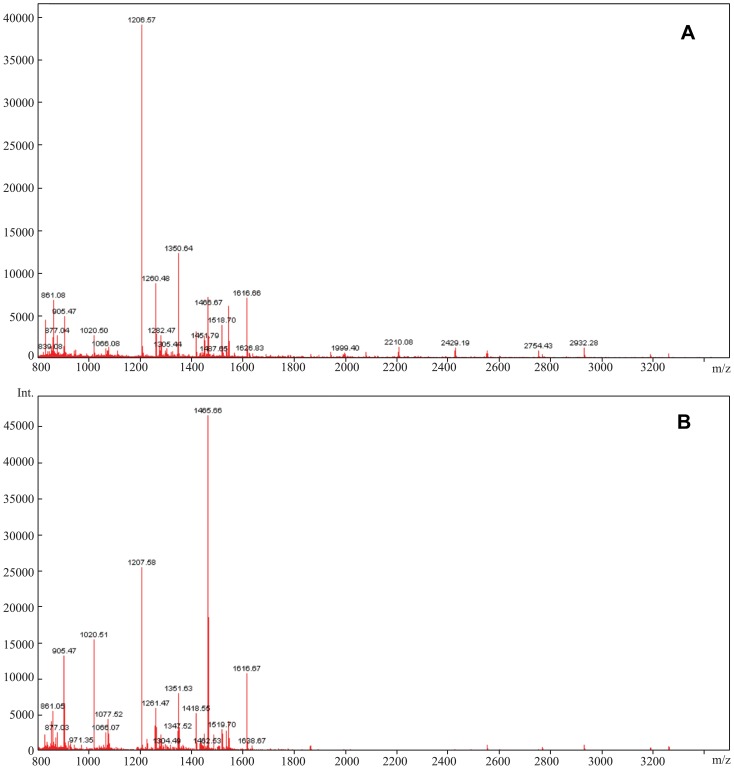
MALDI-TOF MS spectra of one twin pair's peptide profile using C18 resin. (A) MALDI-TOF MS spectrum of the twin in the disease group. (B) MALDI-TOF MS spectrum of the twin in the control group.

**Figure 2:**
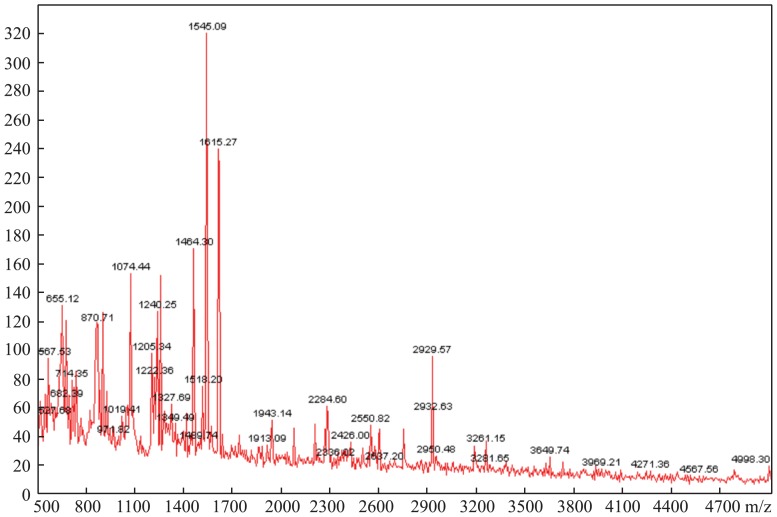
MALDI-TOF MS spectrum of the peptide profile using C4 resin.

Theoretically, ZipTip with C18 resin can bind and purify polypeptide components with a molecular weight less than 5000Da. Of the three methods of affinity chromatography that we tested, PMF with ZipTip with C18 resin (Figure 1) was more effective and reproducible. In addition, the intensity and distribution of the peptides were superior to the other two methods. The abundant mass spectrum peaks were used for the second order mass spectrography to identify the specific amino acid sequences. Figure 2 shows that the amount and intensity of the peptides purified by ZipTip with C4 resin were too low to conduct second order mass spectrography. Though PepClean C18 Spin Columns could abstract peptides from much more serum samples, it resulted in poor MS spectra. Thus, the optimal technique used in this study was affinity chromatography using Ziptip C18 and MALDI-TOF with reflective mode, which resulted in PMFs between 800–3500Da.

### 3 Screening the discriminatory peptides

**Figure 3:**
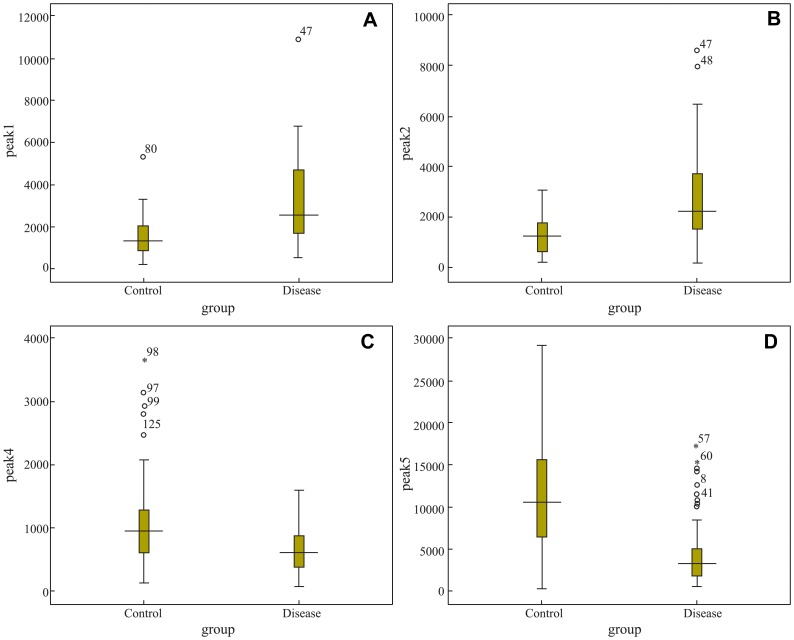
Box-blot of the discriminatory peak signal-intensities. (A), (B), (C), (D) refer to signal-intensities of peaks1, 2, 4 and 5, respectively.

**Figure 4:**
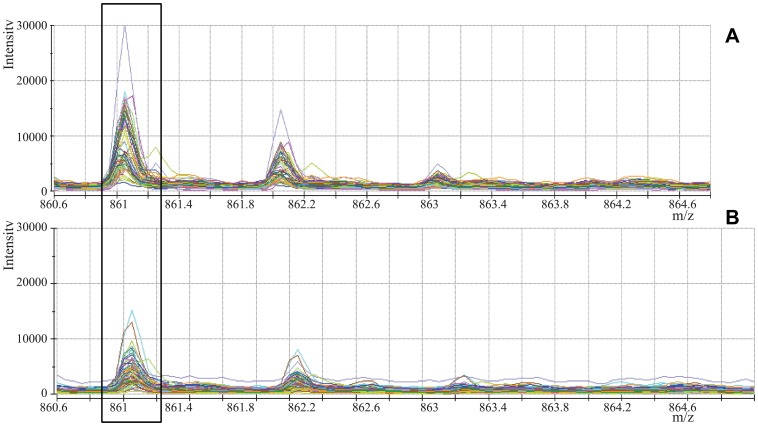
MALDI-TOF MS spectra of the discriminatory peaks. (A) MALDI-TOF MS spectrum of peak1 (861Da) in the disease group. (B) MALDI-TOF MS spectrum of peak1 (861Da) in the control group.

The initial mass spectrum was transferred to ASCII format via Data Explorer software and stored as a peak list file. After comparing the intensities of the same isotopic peaks in the two groups using Metalab software, we screened 18 MS peaks that had significant differences. Among them, 11 peaks (861.1, 877.07, 904.5, 1206.57, 1350.64, 1518.7, 1690.9, 1777.94, 2931.29, 3190.4, 3261.4) were up-regulated, whereas 7 peaks (942.44, 1020.47, 1060.06, 1211.7, 1263.63, 1449.76, 2768.3) were down-regulated in the disease group as compared to the control group. Table 3 shows the mean intensities of the discriminatory peptides and Figure 3 shows the Box-blot of the discriminatory peptide signal-intensities. Mass spectra comparing the signal-intensities of the discriminatory peaks are shown in Figure 4.

**Table 3:** The mean intensities of the discriminatory peaks.

Peaks	Molecular Weight	Mean intensities	
		NAFLD	Control
Peak1*	861.1	3.2290E3	1.5863E3
Peak2*	877.07	2.6700E3	1.2976E3
Peak3*	904.5	4.5305E3	1.4906E3
Peak4	942.44	6.6995E2	1.0869E3
Peak5	1020.47	4.4476E3	1.2251E4
Peak6	1060.06	2.3634E2	4.3490E2
Peak7*	1206.57	2.7648E4	9.3652E3
Peak8	1211.7	3.5049E2	7.6896E2
Peak9	1263.63	1.0948E3	3.8355E3
Peak10*	1350.64	1.0086E4	2.3314E3
Peak11	1449.76	5.0885E2	1.5149E3
Peak12*	1518.7	4.3341E3	1.7353E3
Peak13*	1690.9	3.4106E2	1.1129E2
Peak14*	1777.94	5.2837E2	1.1562E2
Peak15	2768.3	2.1085E2	4.6206E2
Peak16*	2931.29	8.4654E2	3.5836E2
Peak17*	3190.4	2.7906E2	1.3702E2
Peak18*	3261.4	4.1266E2	1.6163E2

*the up-regulated peaks in NAFLD.

### 4 System cluster

**Figure 5:**
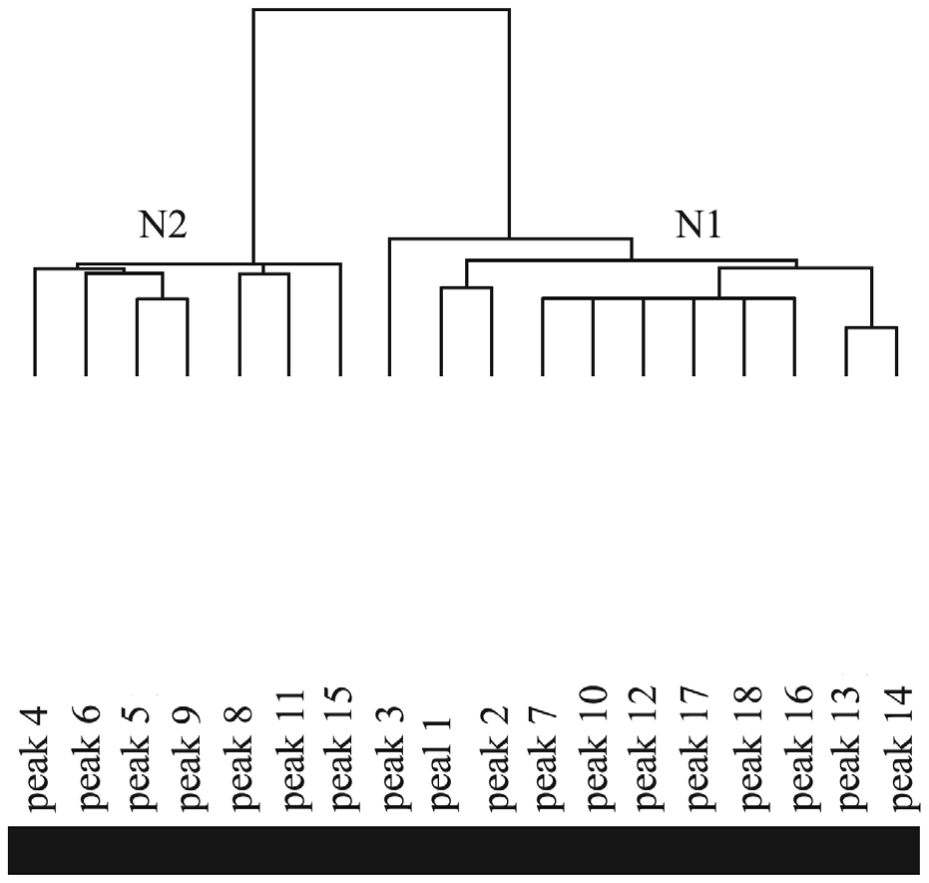
Clustering for variables of the 18 discriminatory peaks. (N1) The 11 up-regulated peaks in the disease group. (N2) The 7 down-regulated peaks in the disease group.

**Figure 6:**
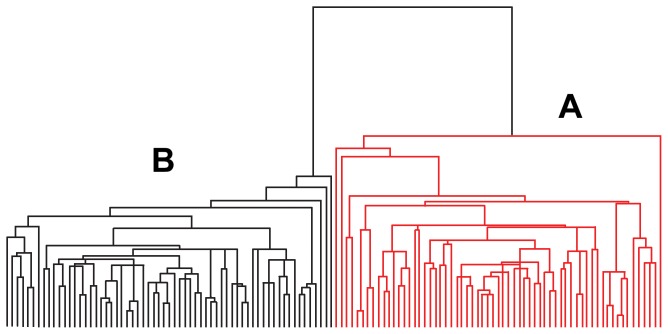
Clustering for individuals of the 126 samples. (A) The 63 disease samples. (B) The 63 control samples.

As shown in the clustering for variables results (Figure 5), the up-regulated peaks (N1 = 11) and the down-regulated peaks (N2 = 7) resulted in 18 discriminatory peptides plit. All of the samples in the disease (A) and normal (B) groups were successfully separated following the clustering for individuals (Figure 6).

### 5 Principal Component Analysis (PCA)

**Figure 7:**
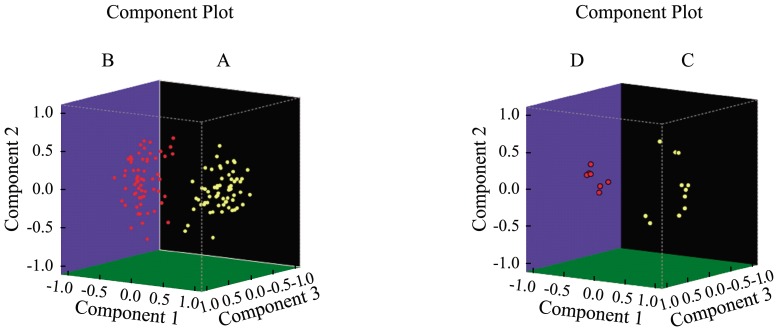
Three-dimensional plot of the principal components of samples and indexes,respectively. (A) Disease group. (B) Control group. (C) The up-regulated peaks. (D) The down-regulated peaks.

Figure 7 showed the principal components of the samples and the principal components of indexes. All 126 samples were clearly classified into two groups: the disease group (A) and the control group (B). Charged with the selected index, the 18 discriminatory peaks were also classified into two groups: the up-regulated peaks (C) and the down-regulated peaks (D). All of the peaks were separated explicitly.

### 6 ROC analysis

**Figure 8:**
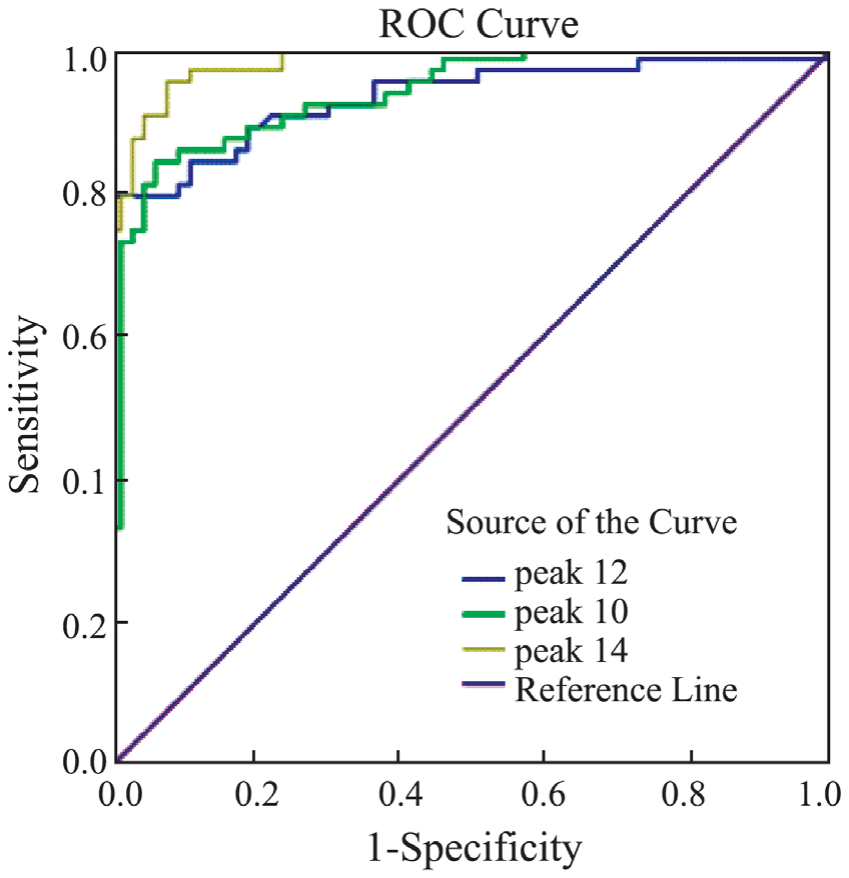
Receiver operated curves of peaks 10, 12 and 14.

For each of the 18 discriminatory peptide peaks, the AUC value was calculated (Table 4). Only the components of peak 10, 12, and 14 were identified successfully by our MALDI-TOF-TOF analysis. Then, for these three discriminatory peaks whose AUC values were higher than 0.9, we conducted ROC curves (Figure 8) to compare their diagnostic efficacy. As shown, peak 14 had the highest diagnostic efficacy.

**Table 4:** Area under the curve of the 18 discriminatory peaks.

	Area	Std. Error^a^	Asymptotic sig^b^	95% CI
Peak1	0.798	0.039	0.000	0.722–0.873
Peak2	0.760	0.043	0.000	0.676–0,843
Peak3	0.794	0.041	0.000	0.714–0.874
Peak4	0.307	0.047	0.000	0.215–0.398
Peak5	0.166	0.037	0.000	0.093–0.238
Peak6	0.166	0.035	0.000	0.097–0.235
Peak7	0.869	0.032	0.000	0.807–0.932
Peak8	0.210	0.039	0.000	0.133–0.286
Peak9	0.066	0.020	0.000	0.026–0.106
Peak10	0.937	0.021	0.000	0.896–0.978
Peak11	0.066	0.025	0.000	0.018–0.115
Peak12	0.931	0.024	0.000	0.884–0.978
Peak13	0.916	0.023	0.000	0.871–0.961
Peak14	0.982	0.008	0.000	0.966–0.999
Peak15	0.109	0.027	0.000	0.056–0.161
Peak16	0.922	0.022	0.000	0.878–0.966
Peak17	0.869	0.030	0.000	0.810–0.928
Peak18	0.911	0.025	0.000	0.862–0.960

a: Under the nonparametric assumption.

b: Null hypothesis: true area =  0.5.

### 7 Identification of the discriminatory peaks

Besides using MALDI-TOF-TOF to trace the sources of discriminatory peptides and their specific amino acid sequences, we also referred to previously published records [24]–[26] (Table 5). Peaks 10, 12, and 14 were up-regulated peaks and corresponded to the peptide family Fibrinopeptide A and Complement C3f, respectively. According to the ROC analysis, Fibrinopeptide A and C3fhad the highest ROC values for distinguishing NAFLD cases from their normal controls.

**Table 5:** Peptide family and amino acid sequences.

	Peptide (m/z)	Peptide family	Amino acid sequence
Peak1	861.1	Unkown	Unkown
Peak2	877.07	Unkown	Unkown
Peak3	904.5	Bradykinin	RPPGFSPF
Peak4	942.44	Complement C3f	HWESASLL
Peak5	1020.47	Fibrinopeptide A	DFLAEGGGVR
Peak6	1060.06	Bradykinin	RPPGFSPFR
Peak7	1206.57	Fibrinopeptide A	EGDFLAEGGGVR
Peak8	1211.7	Complement C3	RIHWESASLL
Peak9	1263.63	Fibrinopeptide A	GEGDFLAEGGGVR
Peak10	1350.64	Fibrinopeptide A	SGEGDFLAEGGGVR
Peak11	1449.76	Complement C3f	THRIHWESASLL
Peak12	1518.7	Fibrinopeptide A	AD_dh_AGEGDFLAEGGGVR _dh_A: dehydro-alanine
Peak13	1690.9	Complement C3f	KITHRIHWESASLL
Peak14	1777.94	Complement C3f	SKITHRIHWESASLL
Peak15	2768.3	Fibrinogen α	SSSYSKQFTSSTSYNRGDSTFESKS
Peak16	2931.29	Fibrinogen α	SSSYSKQFTSSTSYNRGDSTFESKSY
Peak17	3190.4	Fibrinogen α	SSSYSKQFTSSTSYNRGDSTFESKSYKM
Peak18	3261.4	Fibrinogen α	SSSYSKQFTSSTSYNRGDSTFESKSYKMA

### 8 ELISA-based validation experiment for serum Complement C3f and Fibrinopeptide A

**Figure 9:**
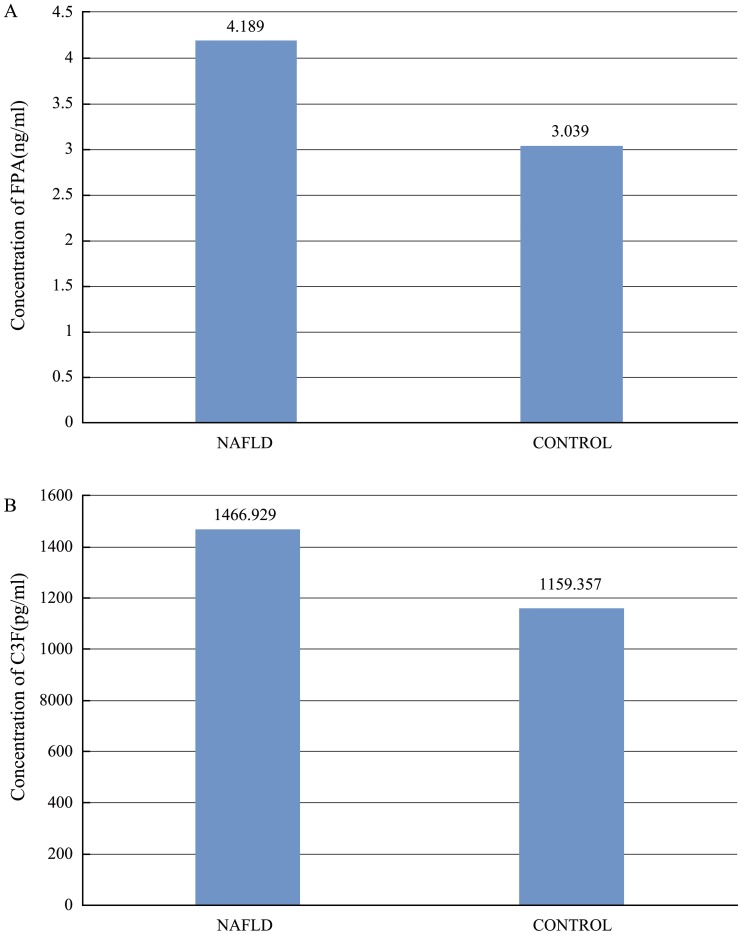
Concentrations of FPA and Complement C3f in NAFLD group and control group, respectively. (A) Concentrations of FPA in NAFLD group and control group. (B) Concentrations of Complement C3f in NAFLD group and control group.

To assess the clinical usefulness of these two candidate biomarkers, we conducted further validation studies by commercially available ELISA kits using 100 additional serum samples (50 NAFLD patients and 50 healthy controls from the Center of Health Examination, Qingdao Municipal Hospital) (Table 2). Prior to ELISA analysis, serum samples were diluted 5-fold with sample dilution, the final volume of test samples was 50 µl, respectively. All procedures were according to the manufacturer's instructions. Spectrometer was used to test the OD values at the 450 µm of the spectrum band. The serum levels of these two candidate biomarker peptides were calculated on the basis of standard curves, respectively. We compared the concentrations of the Complement C3f (1466.929±78.306 pg/ml) and Fibrinopeptide A (4.189±0.326 ng/ml) in NAFLD group with in control group (C3f 1159.357±99.624 pg/ml; FPA 3.039±0.483 ng/ml) by independent-samples T test, respectively. The results revealed that peptides showed significant differences in their serum levels between the two groups (P<0.05) as shown in figure 9. This validation experiment further indicated that FPA and Complement C3f may be potential diagnostic markers for NAFLD.

## Discussion

Although most NAFLD patients are clinically asymptomatic, they are at an increased risk for hepatic fibrosis, cirrhosis and HCC [27], [28]. Liver biopsy represents the traditional gold standard for NAFLD diagnosis [29], [30]. Nevertheless, liver biopsy has limitations due to high cost, sampling error, and possible procedure-related morbidity and mortality [31], [32]. The worldwide prevalence of NAFLD ranges from 6.3% to 33% with a median of 20% [33], and thus liver biopsy would not be a practical and available approach for diagnosing NAFLD in a large population [34]. In addition, noninvasive diagnosis of NAFLD remains difficult. Serum aminotransferase levels and imaging tests such as ultrasound, computed tomography (CT), and magnetic resonance imaging (MRI) are primarily used to assess steatosis and possibly cirrhosis in patients with NAFLD[35], [36];however, they are not completely reliable. Serum aminotransferases levels can remain within normal ranges despite established NAFLD. For examples, NASH was diagnosed in up to 59% of NAFLD patients despite normal ALT levels [37], [38]. Imaging tests lack sensitivity when steatosis affects less than 30% of the hepatocytes [36]. Also, imaging tests are expensive and cumbersome as screening procedures. Therefore, reliable noninvasive methods for assessing the presence of NAFLD are urgently needed. Developing clinical prediction rules and non-invasive biomarkers for identifying NAFLD would represent major advances in the field[39].In this respect, different high throughput ‘‘unbiased’’ approaches for biomarkers identification have emerged [40], [41].

In our study, using a highly sensitive and specific approach which combines MALDI-TOF-MS and bioinformatics analyses [41], we successfully established a peptidomics pattern that could distinguish NAFLD patients from their twin controls. Specifically, our results indicate that complement C3f and Fibrinopeptide A (FPA) are potentially noninvasive biomarkers for identifying patients with NAFLD. Though complement C3f and FPA have been found to be associated with some diseases, such as cognitive dysfunction in elderly patients and breast cancer[42], [43], and complement C3f has been reported to be associated with HCC[44], the association between these two complements and the risk for incidental NAFLD have not yet been reported. Without data concerning the severity (in terms of inflammation and fibrosis) of NAFLD in our patients, the possible role of complement C3f and FPA cannot be completely documented. Further studies, coupled with histological classification, which characterizes NAFLD into simple steatosis, NASH or cirrhosis, need to be conducted.

Despite these shortcomings, our study used two technologies with high technical specificity and sensitivity and was based on a disease-discordant monozygotic twin approach, and thus avoided many confounders. In addition, discordant monozygotic twin studies are a powerful method to identify DNA sequence variants, epigenetic variation and metabolites associated with disease and can aid in the detection of biomarker profiles for medical conditions [45], [46].

## Conclusion

In this study, applying sophisticated bioinformatics tools to analyze the complex data obtained from MALDI-TOF-MS analyses, we established a peptidomics pattern that could distinguish NAFLD patients from their twin controls. The differentially-regulated peptides identified in our study, especially Fibrinopeptide A and Complement C3f, may be potential diagnostic markers or therapeutic targets for NAFLD. However, further studies on the relationship between these biomarkers and NAFLD are warranted
